# The Therapeutic Effect of Pamidronate on Lethal Avian Influenza A H7N9 Virus Infected Humanized Mice

**DOI:** 10.1371/journal.pone.0135999

**Published:** 2015-08-18

**Authors:** Jian Zheng, Wai-Lan Wu, Yinping Liu, Zheng Xiang, Ming Liu, Kwok-Hung Chan, Siu-Ying Lau, Kwok-Tai Lam, Kelvin Kai-Wang To, Jasper Fuk-Woo Chan, Lanjuan Li, Honglin Chen, Yu-Lung Lau, Kwok-Yung Yuen, Wenwei Tu

**Affiliations:** 1 Department of Paediatrics & Adolescent Medicine, University of Hong Kong, Hong Kong, China; 2 State Key Laboratory of Emerging Infectious Diseases, Department of Microbiology, University of Hong Kong, Hong Kong, China; 3 Guangzhou Institute of Respiratory Diseases, Guangzhou Medical University, Guangzhou, China; 4 State Key Laboratory for Diagnosis and Treatment of Infectious Diseases, First Affiliated Hospital, College of Medicine, Zhejiang University, Hangzhou, China; Washington University School of Medicine, UNITED STATES

## Abstract

A novel avian influenza virus H7N9 infection occurred among human populations since 2013. Although the lack of sustained human-to-human transmission limited the epidemics caused by H7N9, the late presentation of most patients and the emergence of neuraminidase-resistant strains made the development of novel antiviral strategy against H7N9 in urgent demands. In this study, we evaluated the potential of pamidronate, a pharmacological phosphoantigen that can specifically boost human Vδ2-T-cell, on treating H7N9 virus-infected humanized mice. Our results showed that intraperitoneal injection of pamidronate could potently decrease the morbidity and mortality of H7N9-infected mice through controlling both viral replication and inflammation in affected lungs. More importantly, pamidronate treatment starting from 3 days after infection could still significantly ameliorate the severity of diseases in infected mice and improve their survival chance, whereas orally oseltamivir treatment starting at the same time showed no therapeutic effects. As for the mechanisms underlying pamidronate-based therapy, our in vitro data demonstrated that its antiviral effects were partly mediated by IFN-γ secreted from human Vδ2-T cells. Meanwhile, human Vδ2-T cells could directly kill virus-infected host cells in a perforin-, granzyme B- and CD137-dependent manner. As pamidronate has been used for osteoporosis treatment for more than 20 years, pamidronate-based therapy represents for a safe and readily available option for clinical trials to treat H7N9 infection.

## Introduction

The first human epidemic of avian-origin influenza virus infection caused by subtype H5N1 occurred in 1997 [1]. In February 2013, a novel avian-origin influenza A virus H7N9 was firstly identified in patients in Eastern China [2–6]. During the first wave of H7N9 infection among human population in 2013, 143 patients with 46 deaths were confirmed in 11 provinces or municipalities in China, leading to the concern of a new influenza pandemic. Although the limited human-human transmission of H7N9 virus decreased the possibility of a global pandemic, the return of H7N9 infection trend in early 2014 aroused the concentrations on improving the control and treatment of newly-occurred avian influenza virus [7]. On the other hand, commercial antiviral drugs such as oseltamivir and zanamivir are generally effective in reducing viral load and improving clinical outcome once being administrated within 48 hours post symptom onset, but are less effective in severe cases who are often presented late [8]. Moreover, H7N9 strains carrying Arg292Lys mutation in neuraminidase [6] gene were identified in two H7N9-infected patients recently and exhibited resistance to the treatment of adamantanes, oseltamivir and zanamivir [9], which made developing alternative therapeutic strategies to treat H7N9 infection in urgent need.

As the first line of host immune defense system, innate immunity plays a critical role in the early defense against influenza A virus infections. More importantly, innate immunity-targeted strategy shows advantage over the virus-targeted therapy in avoiding the failure of treatment due to frequent antigen drift and shift as seen in influenza virus [10]. Although only representing for 1–10% of T lymphocytes in peripheral blood of adult humans and animals, γδ-T cells have been confirmed to be an important component of innate immunity and equipped with broadly antiviral and anti-tumor capabilities [11,12]. In humans, more than 95% of γδ-T cells in peripheral blood and lymphoid organs are Vδ2-T cells. As human Vδ2-T cells could be specifically activated and expanded in an HLA unrestricted manner by small non-peptidic phosphoantigens [13], this minor population of peripheral T lymphocyte represents for a potential target in antiviral and anti-tumor therapy. In previous reports, we have shown that pamidronate, a pharmacological phosphoantigen currently used in treating osteoporosis, exhibited protective effects against influenza infection caused by seasonal H1N1 and avian H5N1 viruses in both in vitro and in vivo models [14–17]. We also demonstrated that the antiviral effects of pamidronate were dependent on Vδ2-T cells and mediated by their cytokine secretion and cytotoxicity against virus-infected host cells [14–17]. However, direct comparison between the efficacies of pamidronate with conventional antiviral drugs is still lack.

In this study, using a fatal H7N9 infection model established on humanized mice, we compared the therapeutic effects of oseltamivir and pamidronate administrated according to their clinical application. It was demonstrated that, compared to oral treatment of oseltamivir, intraperitoneal (i.p.) injection of pamidronate could significantly reduce the severity of H7N9 infection and improve the survival rate of infected mice by enhancing Vδ2-T cell-mediated immunity. More importantly, pamidronate treatment starting 3 days post infection could still decrease disease severity and the mortality, whereas oseltamivir treatment starting at this time frame showed no such therapeutic effect. Meanwhile, we further investigated the molecular mechanisms underlying Vδ2-T cell-mediated antiviral effects in in vitro primary culture system, and found that IFN-γ, perforin, Granzyme B and CD137 expressed by Vδ2-T cell played important roles in this antiviral effect. Our study suggested a new therapeutic option for treating H7N9 virus infection.

## Materials and Methods

### Generation of Humanized Mice

Rag2^−/−^γc^−/−^ mice were purchased from Taconic and maintained in individual ventilated cages (IVC) system under specific pathogens-free (SPF) environment in the Laboratory Animal Unit, the University of Hong Kong. Human PBMCs were isolated from buffy coat preparations of blood from healthy donors collected by the Hong Kong Red Cross with written consent. Humanized mice were established in 4 weeks old Rag2^−/−^γc^−/−^ mice by i.p. transplanting 30×10^6^ human PBMCs as we described previously [17,18]. These chimeric Rag2^-/-^γc^-/-^ mice had functional human T and B cells including similar percentage of circulating Vδ2^+^ T cells compared to human after 4 weeks of PBMC transplantation and thus were referred to as “humanized” mice. All manipulations on animals were performed in compliance with the Animals (Scientific Procedures) Act, 1986 (UK) (amended in 2013) and approved by the Committee on the Use of Live Animals in Teaching and Research (CULATR), Hong Kong (approval number: CULATR 2378–11). All sections of this report adhered to the ARRIVE Guidelines for reporting animal research and a completed ARRIVE guidelines checklist was included in S1 Checklist. All manipulations on human PBMCs were approved by the Institutional Review Board (IRB) of the University of Hong Kong/Hospital Authority Hong Kong West Cluster, Hong Kong (approval number: UW07-154).

### Viruses, Infections, and Treatment of Virus-Infected Humanized Mice

Avian influenza virus H7N9 (A/Zhejiang/DTID-ZJU01/2013) were cultured in SPF eggs and the viral titer was determined by daily observation of cytopathic effect in MDCK infected with serial dilutions of virus stock; median tissue culture infective dose (TCID_50_) was calculated according to the Reed-Muench formula. Humanized mice were separated into corresponding groups based on matched sex, weight, and the source of human PBMCs before infection with H7N9 virus (25μl, 10^6^, 10^4^, and 10^2^TCID_50_ respectively) via intra nasal (i.n.). For intervention studies, a human equivalent dose of pamidronate (Pamisol; Hospira Australia Pty Led) or saline of equivalent volume was injected i.p. on day 1 (10mg/kg body weight) and day 3, 5 (5mg/kg per dose) post infections, while oseltamivir (kindly provided by F.Hoffmann-La Roche Ltd, Basel, Switzerland) or saline of equivalent volume was given by oral gavage at 50mg/kg twice a day for 5 consecutive days since day 0 post infection [19]. For delayed-treatment, Pamidronate were injected i.p. on day 3 (10mg/kg), 5, 7 and 9 (5mg/kg per dose) post infections, while oseltamivir-treated mice were given oseltamivir at 50mg/kg by oral gavage twice a day for 5 consecutive days (day 3–7 post infection). 5–6 mice per group were used for each independent experiment while all experiments were replicated 1–2 times for obtaining unbiased data with the minimum quantity of used animals. Mice with >30% weight loss were sacrificed and counted as death.

In all animal experiments, mice will be monitored twice a day during the whole process, while food and water was placed in cages for them to obtain easily. Meanwhile, soft and clean bedding, quiet environment and circadian light will be provided to reduce animal stress. No unexpected death occurred during this study and mice were euthanized by cervical dislocation under anesthesia (by i.p. injection of ketamine plus xylazine at the final concentration of 7.5mg/kg and 0.88mg/kg respectively) when the study was completed or one of the following conditions was observed: body weight loss>30%; body temperature±>2°C; cardiac/respiratory rate±>50%; signs of severe pneumonia (very weak and pre-comatose). To minimize animal suffering and distress, all invasive manipulations will be carried out under anesthesia as described above.

### Immunohistochemistry Assays of Lungs

The lungs from infected humanized mice were harvested on day 5 or 7 post infection, fixed with 10% formalin, and maintained in 75% ethanol. Paraffin-embedded lung sections were prepared according to standard protocols and stained with hematoxylin and eosin. All lung sections were screened, and five fields of each sample were selected randomly by 2 independent observers for evaluating the levels of pathology among different groups.

### Determination of Virus Copy, Inflammatory Cytokines/Chemokines in Lungs

The lungs from infected humanized mice were harvested on day 1, 3, 5 or 7 post infection and homogenized in 2 ml of PBS. After centrifugation at 1,500 g for 15 min, the supernatants were collected for the determination of viral load and inflammatory cytokines, chemokines. The concentrations of human cytokines and chemokines were detected and analyzed with human cytokine and chemokine assay kits (Bender MedSystems).

### Generation of Pamidronate-Expanded Vδ2-T Cells and MDMs

Human PBMCs were cultured in 10% FBS RPMI-1640 medium with 9μg/ml of pamidronate. Recombinant human IL-2 (Invitrogen) was added into the medium at the final concentration of 500IU/ml every 3 d from day 3. After 14-d culture, Vδ2-T cells were purified by positive selection with anti-human TCR γ/δ^+^ T cell microbeads according to the manufacturer’s instruction (Miltenyi Biotec). Human Monocyte-derived macrophages (MDMs) were generated from monocytes. Briefly, adherent monocytes were cultured in RPMI-1640 supplemented with 5% autologous serum and allowed to differentiate to macrophages for 14 d.

### Cytotoxic Assay

Human MDMs (Target, T) were stained with Carboxyfluorescein succinimidyl ester (CFSE) and infected with H7N9 influenza virus at multiplicity of infection (MOI) of 2 before co-culturing with autologous Vδ2-T cells (Effector, E) at different E:T ratios for 6 h. Cells were then stained with ethidium homodimer-2 (EthD-2) to identify dead ones. The cytotoxicity of Vδ2-T cells against virus-infected MDMs was assessed by flow cytometry as the percentage of EthD-2^+^ cells in CFSE^+^ population with FACSAria (BD) and FlowJo software (Tree Star) (S1 Fig).

### Quantification of Viral Copies by RT-PCR

Human MDMs were infected by H7N9 virus at MOI of 2. 1 h later, un-adsorbed virus was washed away carefully and MDMs were cultured alone or with Vδ2-T cells for 48 h. Then the cells and supernatant were harvested for extraction of total RNA by TRIzol LS reagent according to the manufacturer’s instructions (Invitrogen). The cDNA was synthesized with oligo (dT)_12-18_ primer and Superscript II reverse transcription (Invitrogen). Viral matrix gene copies were quantified on the basis of SYBR green fluorescence signal after real-time PCR procedure (forward primer, 5′-CTTCTAACCGAGGTCGAAACG-3′; reverse primer, 5′-GGCATTTTGGACAAAGCGTCTA-3′) by ABI PRISM 7900 Sequence Detection System (Applied Biosystems). Results were expressed as the number of target gene copies per 10^3^ MDMs.

### Blocking Assay

Vδ2-T cells were co-cultured with H7N9 virus–infected MDMs at indicated E:T ratio. The neutralization antibodies mouse anti-human NKG2D (monoclonal; clone number: 1D11; BD), mouse anti-human FasL (monoclonal; clone number: 100419; R&D Systems), mouse anti-human TRAIL (monoclonal; clone number: RIK-2; R&D Systems), mouse anti-human CD137 (monoclonal; clone number: 4B4-1, Biolegend), mouse anti-human CD244 (monoclonal; clone number: 2–69; BD), goat anti-human IFN-γ (polyclonal; R&D Systems) and their relevant isotype controls (IC) were used at the final concentration of 10μg/ml. For blocking perforin and granzyme B, MA (Sigma-Aldrich) and Bcl-2 (R&D Systems) were used respectively. The cytotoxicity were then calculated by CFSE and EthD2 staining as described previously.

### Statistical Analyses

Data are presented by means ± SEM. Multiple regression analysis was used to test the differences in the body weight changes between different groups adjusted for time after infection. The differences in cytotoxicity and virus copy for in vitro experiments, and viral load or concentrations of pro-inflammatory cytokines/chemokines were analyzed by unpaired two-tailed Student’s *t* test. The P value of the difference for survival was determined by Kaplan-Meier log-rank test. P<0.05 was considered to be significant.

## Results

### Avian Influenza A H7N9 Virus Caused Severe Disease in Humanized Mice

**Fig 1:**
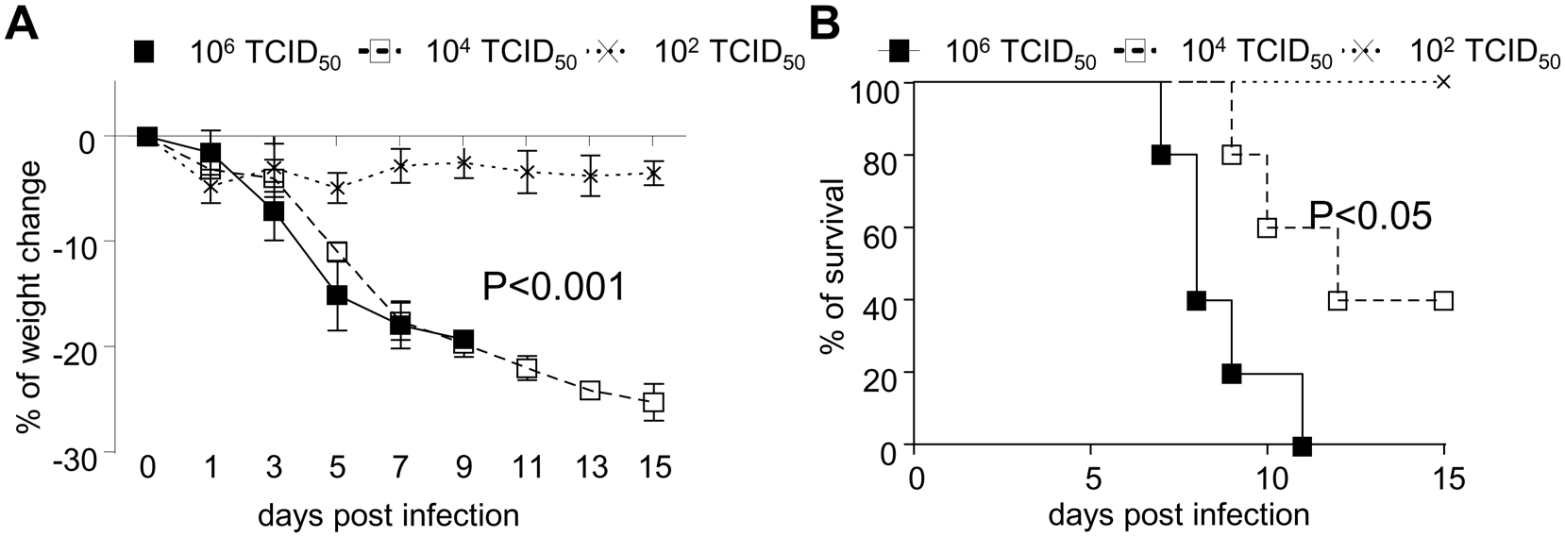
Induction of severe H7N9 infection in humanized mice. Humanized mice were infected with H7N9 virus i.n. at different dose (10^6^, 10^4^, 10^2^ TCID_50_) in 25μl of saline solution on day 0. The weight change (A) and survival (B) in virus-infected mice (5 mice per group) were monitored and recorded till day 15 post infection.

We firstly examined the pathogenic effects of H7N9 virus by i.n. infecting humanized mice with different doses of H7N9 virus. As shown in Fig 1, low dose (10^2^TCID_50_) of virus only induced mild disease in humanized mice with evidence of 5–10% weight loss post infections. In contrast, medium (10^4^TCID_50_) or high (10^6^TCID_50_) doses of virus caused rapid, severe and irreversible weight loss in humanized mice (Fig 1). 33.3% of mice in medium dose of virus-infected group could survive through 15 days post infection, while mice in high dose infection group started to die from day 3 and all mice died within 12 days post infection (Fig 1). We thus chose 10^6^TCID_50_ (high dose) as the dose for establishing severe and fatal H7N9 influenza virus challenge model for the following experiments.

### Pamidronate Exhibited Superior Effects on Controlling H7N9 Infection in Humanized Mice Compared to Oseltamivir

**Fig 2:**
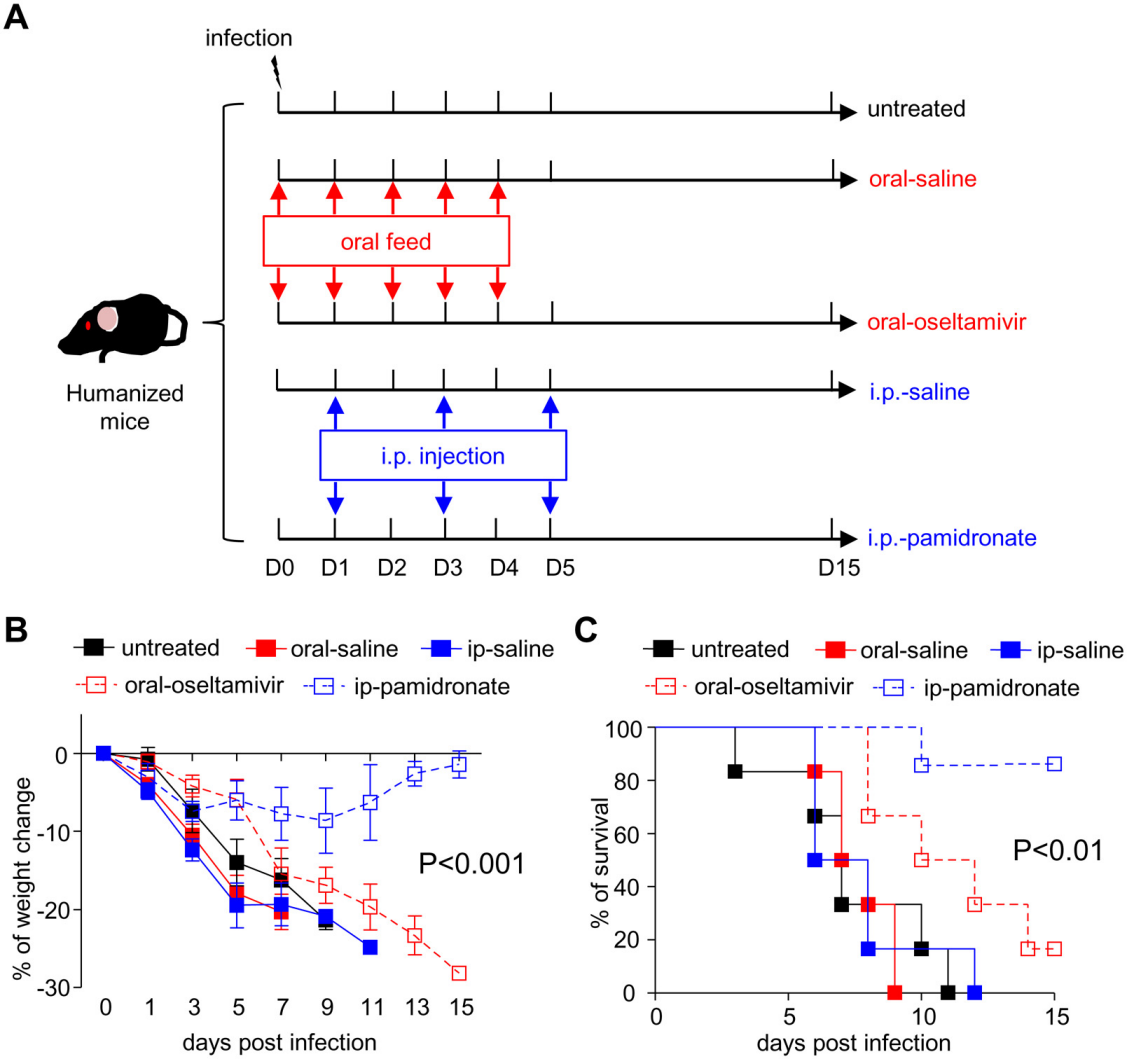
Effects of oseltamivir and pamidronate on lethal H7N9 infection in humanized mice. A. Protocol of assessing effects of oseltamivir and pamidronate on lethal H7N9 infection in humanized mice. Humanized mice established from PBMCs of same donors were infected with H7N9 virus i.n.at 10^6^ TCID_50_ in 25μl of saline solution on day 0 and distributed to corresponding groups. Pamidronate (i.p.-pamidronate group) or saline of equivalent volume (i.p.-saline group) was injected i.p. on day 1 (10mg/kg), 3 and 5 (5mg/kg per dose) post infection whereas 50mg/kg oseltamivir (oral-oseltamivir group) or saline of equivalent volume (oral-saline group) was given by oral gavage twice a day for 5 consecutive days since day 0 post infection. The weight change (B) and survival (C) of virus-infected mice (6 mice per group) were monitored and recorded till day 15 post infection. The data are representative of three independent experiments.

To compare therapeutic effects of pamidronate and oseltamivir on H7N9-infected humanized mice, pamidronate-treated mice were i.p. injected with one full dose drug (10mg/kg) on day 1 post infection, and one half dose (5mg/kg) on day 3 and 5 respectively, while oseltamivir-treated group was given drug by oral gavage twice a day (50mg/kg per dose) for 5 consecutive days (day 0–4 post infection). Saline of same volume was given as control by respective ways (Fig 2A). As shown in Fig 2B and 2C, all mice in untreated group and saline-treated groups had comparably rapid and irreversible weight loss and death within 12 days post virus infection. Although the treatment of oseltamivir ameliorated the fast weight loss of infected mice during the early phase of infection, no significant improvement in survival of infected mice could be obtained. In contrast, treatment with pamidronate significantly improved the prognosis of infected humanized mice as evidenced by decreased weight loss and mortality. These results demonstrated that pamidronate could effectively ameliorate disease severity and the mortality caused by H7N9 virus compared to oseltamivir.

**Fig 3:**
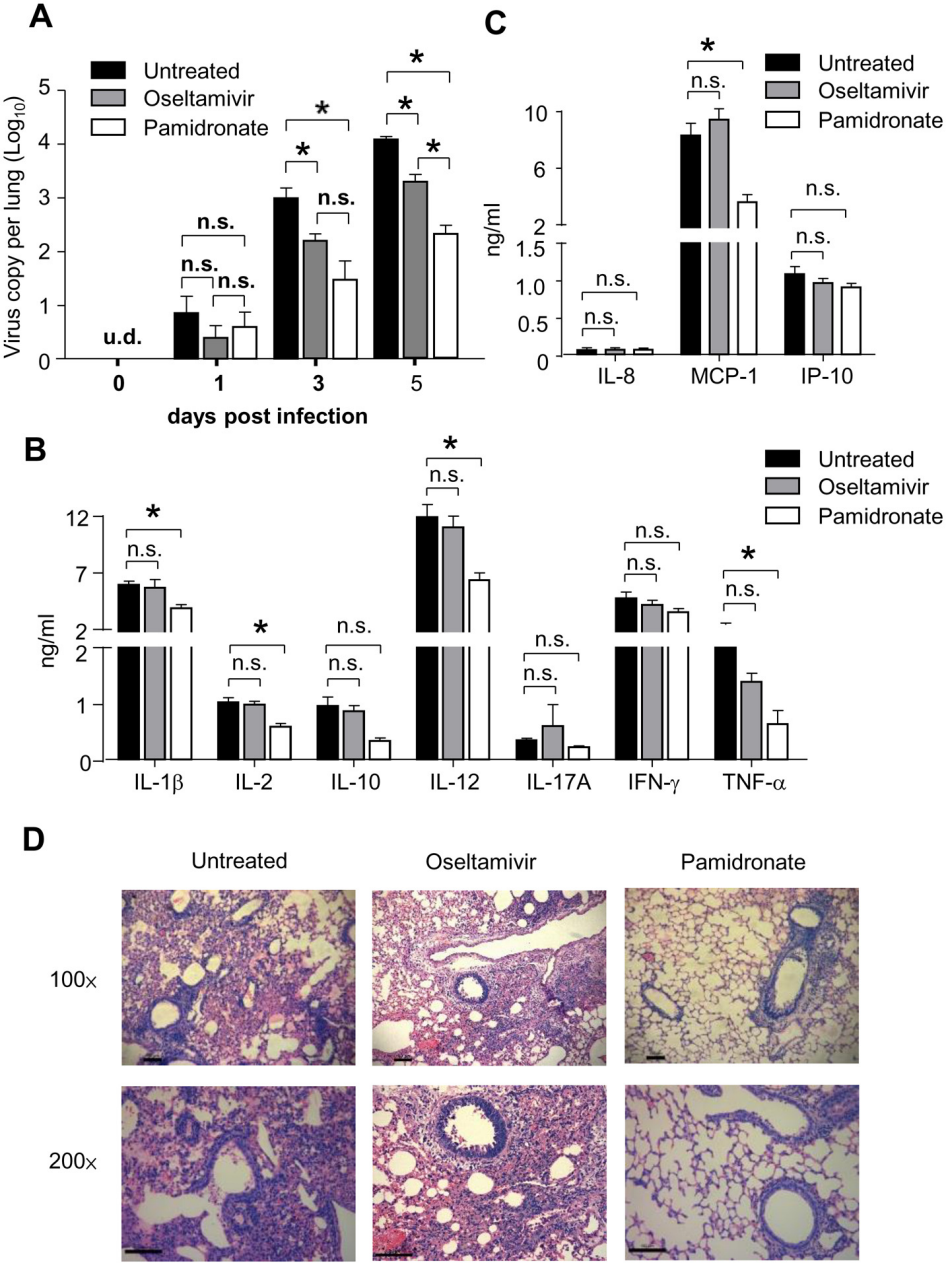
Pamidronate exhibited superior effects in controlling virus replication and inflammation in lungs of H7N9-infected humanized mice. Humanized mice were infected and treated as described previously, while viral loads in the lungs will be determined on day 0, 1, 3, and 5 post infection (A). On day 5 post infection, lungs of H7N9-infected humanized mice from untreated, i.p.-pamidronate and oral-oseltamivir group were harvested and the levels of human pro-inflammatory cytokines (B) and chemokines (C) in the supernatants of homogenized lungs were determined (n = 5). *p<0.05; n.s., no significance; detection limits: IL-8: 14~10,000pg/ml; MCP-1: 55~40,000pg/ml; IP-10: 17~12,500pg/ml; IL-1β, IL-2, IL-10, IL-12, IFN-γ, and TNF-α: 27~20,000pg/ml; IL-17A: 14–10,000pg/ml. (D) Representative histological sections of the lung tissues from H7N9 virus-infected mice receving pamidronate or saline treatment were stained with hematoxylin and eosin. Bars, 100μm. The data are representative of three independent experiments.

To illustrate molecular mechanisms underlying superior performance exhibited by pamidronate compared to oseltamivir, we examined the viral load, pro-inflammatory cytokines and chemokines in the lungs of mice from pamidronate-treated, oseltamivir-treated and untreated groups. As shown in Fig 3A, although both pamidronate and oseltamivir treatment reduced the viral load in the lungs compared to untreated group, viral load in the lungs of pamidronate-treated mice was significantly lower than that of oseltamivir-treated mice on day 5 post infection. Moreover, pamidronate treatment significantly reduced the levels of human IL-1β, IL-2, IL-12, TNF-α and MCP-1 in the lungs as compared to other treatments (Fig 3B and 3C). Furthermore, less infiltration of leukocytes and moderate pathology were observed in the lungs of pamidronate-treated humanized mice on day 5 post-infection compared with that of untreated mice, whereas oseltamivir treatment exhibited no effects on controlling severe lung inflammation caused by infection (Fig 3D). Taken together, these results indicated that pamidronate treatment could inhibit H7N9 virus replication in the lung and attenuate the lung inflammation and pathology, thus exhibited superior potential in controlling H7N9 infection in humanized mice compared to oseltamivir.

### Pamidronate Exhibited Better Therapeutic Effects than Oseltamivir by Delayed Treatment

**Fig 4:**
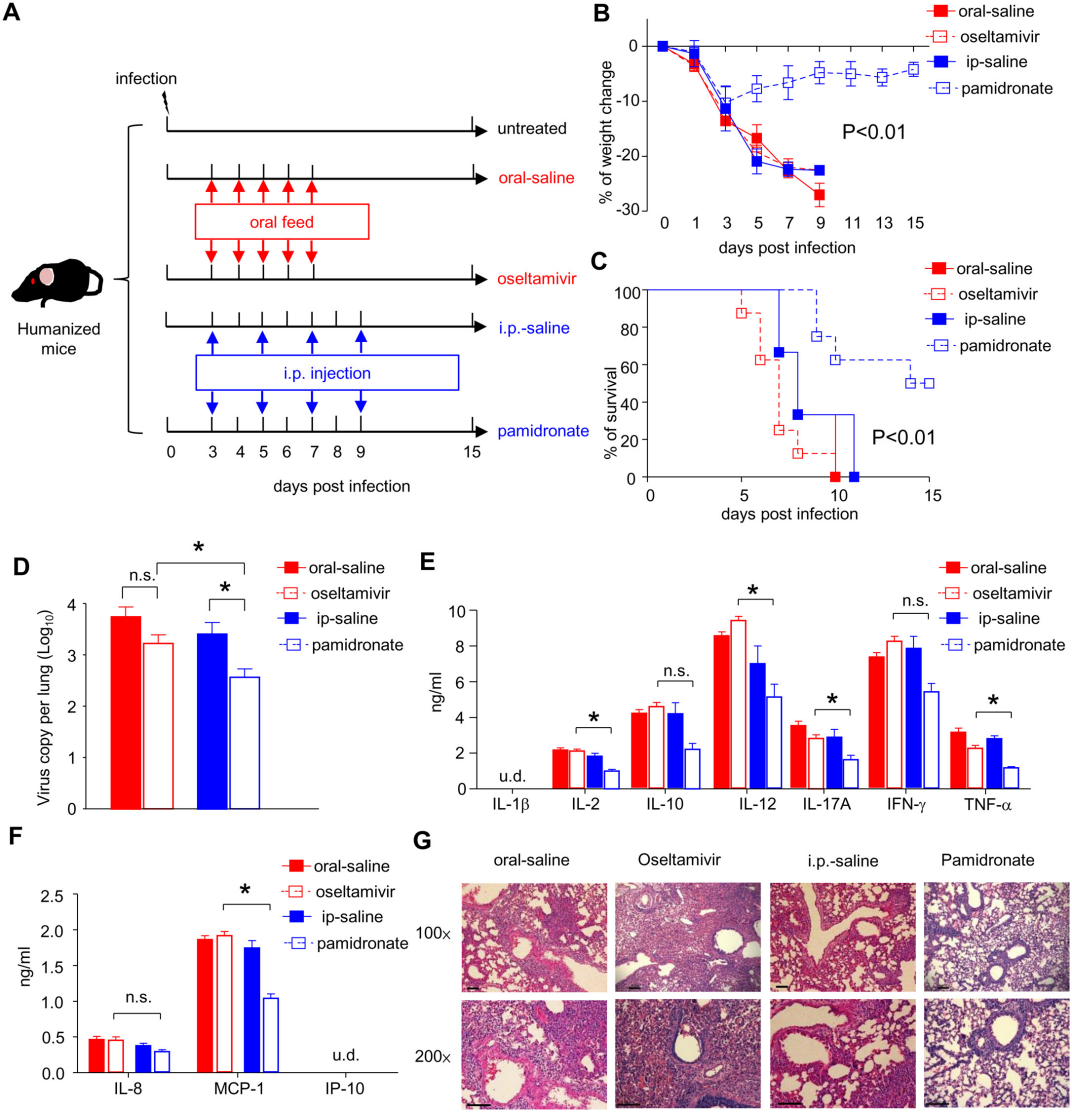
Therapeutic effects of Pamidronate and oseltamivir by delayed treatment. Humanized mice were infected with H7N9 virus i.n. at 10^6^ TCID_50_ in 25μl saline solution on day 0. Pamidronate were injected i.p. on day 3 (10mg/kg), 5, 7 and 9 (5mg/kg per dose) post infection whereas control mice were fed with oseltamivir by oral gavage daily for 5 consecutive days (day 3~7, 50mg/kg each dose) (A). The weight change (B) and survival (C) of infected mice (8 mice for pamidronate treatment group, 8 mice for oseltamivir treatment group, 6 mice for each saline-treated group) were monitored and recorded till day 15 post infections. On day 7 post infection, lungs from different groups were harvested and the viral loads (D) and the levels of human pro-inflammatory cytokines (E) and chemokines (F) in the supernatants of homogenized lung tissue were determined (n = 5). *p<0.05; n.s., no significance; u.d., undetectable; detection limits: IL-8: 14~10,000pg/ml; MCP-1: 55~40,000pg/ml; IP-10: 17~12,500pg/ml; IL-1β, IL-2, IL-10, IL-12, IFN-γ, and TNF-α: 27~20,000pg/ml; IL-17A: 14–10,000pg/ml. (G) Representative histological sections of the lung tissues from H7N9 virus-infected mice receiving pamidronate or oseltamivir treatment were stained with hematoxylin and eosin. Bars, 100μm. The data are representative of three independent experiments.

Since the onset of symptoms in H7N9-infected patients generally occurred on 2–3 days post initial infection, we further evaluated the efficacy of pamidronate versus oseltamivir in humanized mice with severe diseases while being administrated at delayed time frame (Fig 4A). In concert with clinical situation, H7N9-infected humanized mice exhibited abrupt weight loss (approximately 15% of original weight) accompanied with intense inflammation and virus replication in lungs on day 3 post infection (Fig 4B and S2 Fig). Similar to early-treated mice, late pamidronate-treated group was given one full dose drug (10mg/kg) on day 3 post infection and one half dose (5mg/kg) on day 5, 7, and 9 respectively, while oseltamivir-treated group was given oseltamivir orally twice a day (50mg/kg per dose) for 5 consecutive days (day 3–7 post infection). As shown in Fig 4B and 4C, all mice in saline-treated group and oseltamivir-treated group continuously lost their weight and died within 10 days post infection, and there were no significant differences in weight loss and survival among these groups. In contrast, in pamidronate-treated group, the weight loss of mice was ameliorated significantly and 50% of mice recovered during 15 days post infection. These data suggested that pamidronate might be more potent in treating severe influenza disease caused by H7N9 virus compared to oseltamivir, especially for those who presented symptoms late.

To directly compare the efficacy of delayed pamidronate and oseltamivir treatment on virus replication and lung inflammation, we also determined the viral load, pro-inflammatory cytokines and chemokines, and pathology in the lungs. Consistent with that seen in early treatment groups (Fig 3), H7N9 viral loads in the lungs from pamidronate-treated mice were significantly lower than those in the lungs from oseltamivir-treated mice (Fig 4D). Meanwhile, pamidronate treatment significantly reduced the levels of human IL-2, IL-12, IL-17A, TNF-α and MCP-1 in the lungs compared to oseltamivir treatment (Fig 4E and 4F). Finally, there were fewer infiltrated leukocytes and less pathology in the lungs from pamidronate-treated mice on day 7 after H7N9 virus infection compared with that of oseltamivir-treated mice (Fig 4G).

### Cellular and Molecular Mechanisms Underlying Protective Effects of Pamidronate

**Fig 5:**
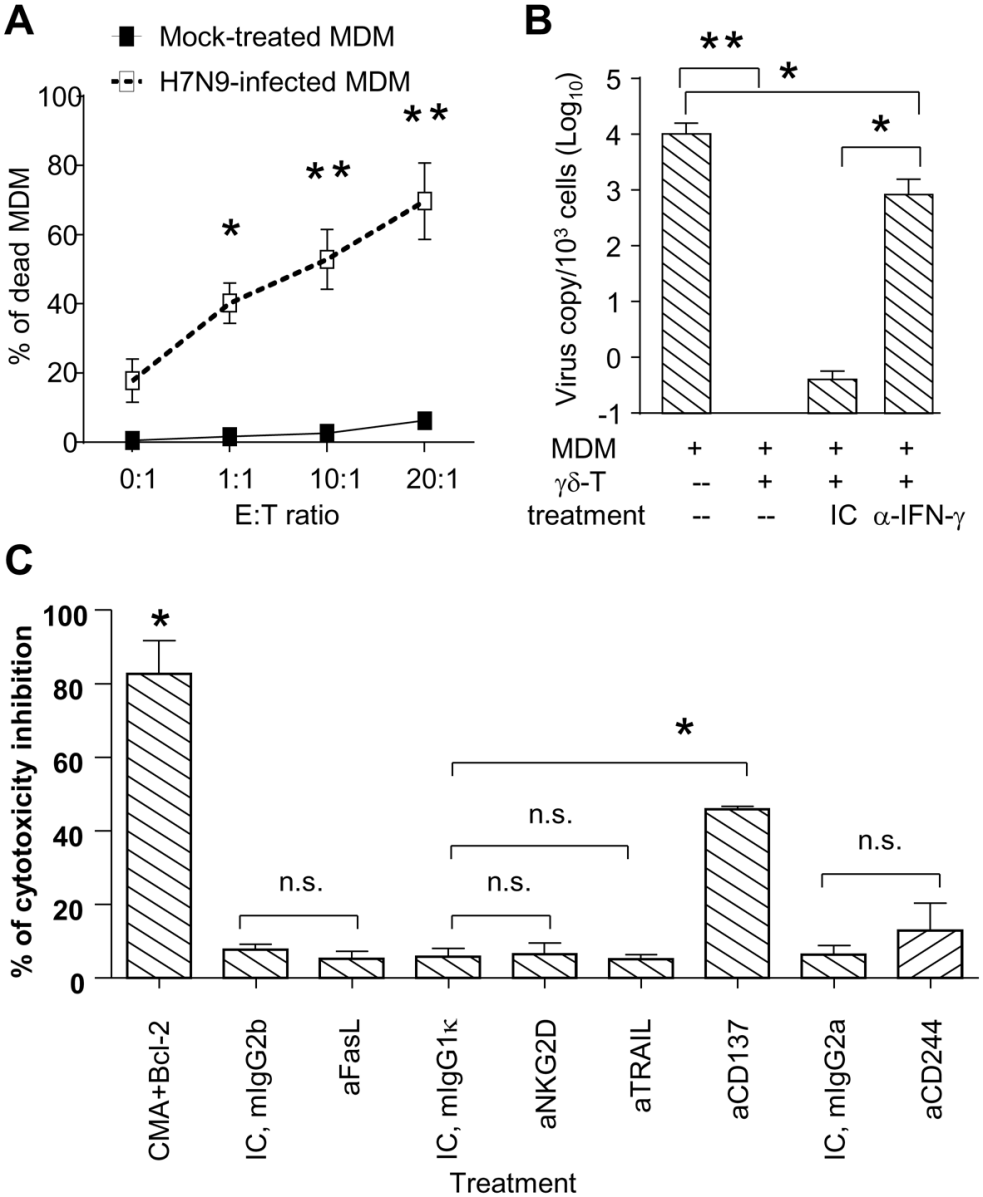
Pamidronate-expanded Vδ2-T cells exhibited both non-cytolytic and cytotoxic antiviral activities against H7N9 virus infection. Human MDMs (target, T) were infected with H7N9 virus or mock at MOI of 2 for 1 hour, and then co-cultured with pamidronate-expanded autologous Vδ2-T cells (effector, E) at indicated E:T ratios for 6 hours. The cytotoxicity of Vδ2-T cells was determined by EthD2 staining on dead MDM at the final 15 minutes of co-culture (A). To determine whether IFN-γ released from Vδ2-T cells was involved in the inhibition of viral replication, Vδ2-T cells and virus-infected MDMs were co-cultured at 1:1 of E:T ratio for 48 hours in the presence of IFN-γ neutralizing antibody (10μg/ml) and its isotype control (IC, goat IgG) respectively. The virus copies (B) in the co-culture were then determined by qPCR (n = 4). (C) Pamidronate-expanded Vδ2-T cells were co-cultured with virus-infected MDMs at a E:T ratio of 10: 1 for 6 h. The perforin inhibitor CMA and granzyme B inhibitor Bcl-2 (both at 1μg/ml), anti-NKG2D (aNKG2D), anti-TRAIL (aTRAIL), anti-FasL (aFasL), anti-CD137 (aCD137), anti-CD244 (aCD244) blocking antibodies (all at 10μg/ml), or their relevant isotype controls were added into the culture since the initiation of incubation. The death of virus-infected MDMs was analyzed by flow cytometry. *p<0.05; **p<0.01; n.s., no significance.

Previously we have demonstrated that the control of influenza virus infections by pamidronate was mediated by human Vδ2-T cells in both cytolytic and non-cytolytic manners [13,17]. To clarify the mechanisms underlying the antiviral activity of pamidronate, pamidronate-expanded human Vδ2-T cells were co-cultured with H7N9 virus-infected autologous human MDMs. As shown in Fig 5A and 5B, Pamidronate-expanded Vδ2-T cells exhibited potent cytotoxic activity against H7N9 virus-infected MDMs and significantly inhibited virus replication in MDMs. Using neutralizing antibodies against IFN-γ, we found that the inhibition of H7N9 virus replication was partially but significantly abrogated by anti-IFN-γ neutralizing antibody (Fig 5B), indicating that pamidronate-expanded Vδ2-T cells could inhibit H7N9 virus replication in host cells in a IFN-γ-dependent manner. Consistent with our previous report, the cytotoxic activities of Vδ2-T cells against H7N9 virus-infected MDMs were significantly abrogated by the combination of CMA and Bcl-2 treatment [16]. Interestingly, anti-CD137, instead of anti-TRAL, anti-FasL, anti-NKG2D or anti-CD244, blocking antibody treatment exhibited significant inhibition on Vδ2-T cell-mediated cytotoxicity (Fig 5C). Taken together, our data demonstrated that pamidronate-expanded Vδ2-T cells could exhibit both non-cytolytic and cytotoxic antiviral activities against H7N9 virus.

## Discussion

Humanized mice model, as a powerful and cost-effective animal model, has been widely used for studying human innate and adaptive immunity against infectious diseases [20,21]. As introduced previously [17], the humanized mice established in our lab by transplantation with human PBMCs were equipped with functional human CD4^+^, CD8^+^ T cells, NK cells and B cells, which could sustain over one year. More importantly, these mice contained a similar percentage of Vδ2-T cells in peripheral blood compared to that in humans [17]. As there are no Vδ2-T cells in mice or ferret, humanized mice provide a satisfactory translational platform for investigating human Vδ2 T cell-mediated immune response in vivo. Meanwhile, we also have confirmed that influenza virus could efficiently replicate in the respiratory system and cause pathology in humanized mice [17]. In this study, by using humanized mice model, we established a H7N9 infection model accompanied with typical characteristics of serious or fatal human influenza infection including severe inflammation and infiltration of leukocytes, intense expression of pro-inflammatory cytokines and chemokines in the lung, rapid weight loss and death. Compared with other investigations using normal mice [22], pigs and ferrets [23] as animal model, humanized mice might represent for better models in exploring novel immune therapy against severe H7N9 infection in human.

Although the benefits of human Vδ2-T cells in defense against some virus infections have been confirmed in clinical and experimental studies, their applications in treating infectious diseases were hampered by their scarcity in circulation [11,24–26]. Phosphoantigen, a small compound produced through mevalonate pathway that could specifically expand and activate Vδ2-T cells both in vitro and in vivo, made Vδ2-T cell-based therapy possible. In this study, we demonstrated that pamidronate, a commercially pharmacological phosphoantigen that has been commonly used for the treatment of osteoporosis for more than two decades [11,24], could effectively control H7N9 virus-caused severe disease in humanized mice through inhibiting virus replication and ameliorating inflammation in the affected lungs. This beneficial effect was mediated by boosting human Vδ2-T-cell-mediated immunity, because no such protective effect could be identified in Vδ2-T cell-deficient humanized mice [17]. Indeed, the ability of pamidronate to control disease caused by H7N9 influenza virus was found to largely depend on the initial quantity of Vδ2-T cells in peripheral blood of humanized mice before therapy. As shown in S3 Fig, One H7N9 virus-infected humanized mice which had low percentage of Vδ2-T cells (<1% of circulating T lymphocytes) eventually died on day 10 post infection despite receiving pamidronate treatment since day 1 post infection. In contrast, other H7N9 virus-infected humanized mice (>1% of circulating T cells being Vδ2-T cells) all recovered after the same treatment with pamidronate, which supported the indispensable role of Vδ2-T cells in pamidronate treatment and indicated that the initial number of Vδ2-T cells might be an important index to evaluate the prognosis of pamidronate-treated patients.

Consistent with previous reports [14,16], here we demonstrated that Vδ2-T cells could exhibit IFN-γ-mediated non-cytolytic ability in inhibiting virus replication. Meanwhile, we also showed that Vδ2-T cells could directly kill H7N9 virus-infected cells, and their cytolytic function was mainly mediated by perforin-granzyme B pathway. Interestingly, it was found that instead of TRAIL, FasL and NKG2D, which were found to be involved in cytotoxicity against seasonal H1N1 and H9N2-infected host cells [13,17], CD137 played an important role in Vδ2-T cell-mediated cytotoxicity against H7N9-infected MDMs. Although these results could not be directly translated into the mechanisms underlying Vδ2-T cell-mediated protection on H7N9-infected humanized mice due to species gap, they undoubtedly improved our understanding on distinct pathways applied by immune system against different influenza virus infections and provided some novel targets for designing specific therapy against unique influenza virus infection.

Resistance to both oseltamivir and zanamivir has been reported for H7N9 virus strains [9,27]. In addition, traditional antivirals are less effective in severe cases, especially when the administration of antiviral therapy was delayed [8]. More recently, clinical meta-analysis also suggested that oseltamivir had only limited effect on clinical symptoms and did not reduce hospitalization or serious complications of influenza infections [28]. Distinct from these traditional strategies, the treatment of pamidronate could expand and activate Vδ2-T cells rather than targeting the virus per se, and thus reduced the emergence of drug-resistant strains. In this study, by directly comparing efficacies of pamidronate versus oseltamivir administrated according to their clinical application, we showed that i.p. injection of pamidronate exhibited better effects in controlling viral replication and inflammatory damage caused by H7N9 virus than oseltamivir fed by oral gavage. As shown in Fig 2, although the oseltamivir treatment could ameliorate the weight loss of infected mice during the early phase (Day 0 to 5 post infection), it could not reverse the final outcome of infected humanized mice. Actually, the inflammation in lungs of oseltamivir-treated mice had been severe on day 5 post infection in spite of lower viral load in lungs compared to that of untreated mice and similar weight loss compared to that of pamidronate-treated mice (Figs 2 and 3), which suggested that even preventive treatment of oseltamivir had only marginal effects on controlling H7N9 virus infection. In contrast, pamidronate exhibited apparent therapeutic effects on H7N9 infection even being given since a late time points (starting from day 3 post infection) (Fig 4), which strongly supported their potential in treating confirmed patient in clinic.

Besides NA inhibitors family, ventilatory support, extracorporeal membrane oxygenation [9,29] and systemic NA inhibitors treatment combined with ribavirin or protease inhibitor aprotinin represented for key treatment modalities in treating H7N9 infections in clinical practice. However, the side effects of ribavirin and aprotinin were prohibitive to their clinical use [30,31]. On the other hand, the efficacy of typical antivirals by sialidase DAS181 [32] might be largely attenuated in established pneumonic consolidation following severe inflammation. Although treatments with high titer specific neutralizing antibodies collected and purified from convalescent-phase plasma or intravenous immunoglobulin appeared effective for treating H1N1 of 1918, 2009 and H5N1 infections, convalescent H7N9 donors that could be recruited are still lacking [33,34]. Non-specific anti-inflammatory recipes such as steroids, statins, and macrolides could decrease the pathology in infected lungs, but might dampen the immune system of patients as well [35]. Finally, delayed treatment with a combination of Cox2 inhibitors and zanamivir was shown to improve survival in A (H5N1) infected mice model [36], but its application in human still need to be evaluated in clinic. Compared to above strategies, Vδ2-T cell-targeted pamidronate treatment exhibited satisfactory control in both viral replication and inflammatory damage, which made it a more promising strategy in future clinical practice.

In conclusion, our study demonstrated that pamidronate could control H7N9 virus infection in humanized mice even by delayed treatment. As pamidronate has been commonly used for treating osteoporosis in clinic for over 20 years, this ‘new application of an old drug’ strategy potentially offers a safe and readily available option for the treatment of H7N9 virus infection.

## Supporting Information

S1 Checklist Completed ARRIVE Guidelines checklist for reporting animal research. (DOCX)

S1 Fig Gating strategy of flow cytometry data on cytotoxicity assay. Total cells in the co-culture of Vδ2-T cells and H7N9 virus–infected MDMs were sorted by forward scatter (FSC) and side scatter (SSC) firstly. MDMs were then gated as CFSE^+^ population, in which EthD-2^+^ cells represented for dead target cells killed by Vδ2-T cells.
(TIF)

S2 Fig Inflammation and virus copy in humanized mice on day 3 post infection with H7N9. Humanized mice were infected with H7N9 virus i.n.at 10^6^ TCID_50_ in 25μl saline solution on day 0. On day 3 post infection, lungs from virus-infected mice were harvested and the levels of pro-inflammatory cytokines (A) and chemokines (B) and the viral loads (C) in the supernatants of homogenized lung tissue were determined (n = 3). Representative histological sections of the lung tissues from H7N9 virus-infected mice on day 3 post infection were stained with hematoxylin and eosin. Bars, 100μm. The data are representative of three independent experiments.
(TIF)

S3 Fig The correlation of initial quantity of peripheral Vδ2-T cells with the weight loss and survival of humanized mice. Pamidronate-treated humanized mice as shown in Fig 2 were grouped according to the initial percentage of Vδ2-T cells in peripheral CD3^+^ T cells. Their weight change (A) and survival (B) were shown here.
(TIF)
