# Supplementary material for: The Therapeutic Effect of Pamidronate on Lethal Avian Influenza A H7N9 Virus Infected Humanized Mice
Source: PLoS One. 2015 Aug 18;10(8):e0135999. doi: 10.1371/journal.pone.0135999 (PMC4540487; doi:10.1371/journal.pone.0135999)

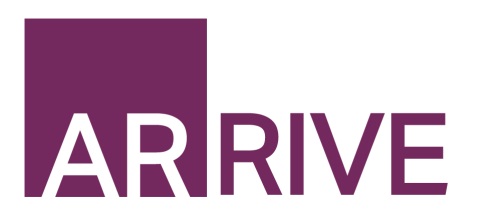


The ARRIVE Guidelines Checklist

**Animal Research: Reporting In Vivo Experiments**

Carol Kilkenny^1^, William J Browne^2^, Innes C Cuthill^3^, Michael Emerson^4^ and Douglas G Altman^5^

*^1^ The National Centre for the Replacement, Refinement and Reduction of Animals in Research, London, UK, ^2^ School of Veterinary Science, University of Bristol, Bristol, UK, ^3^ School of Biological Sciences, University of Bristol, Bristol, UK, ^4^ National Heart and Lung Institute, Imperial College London, UK, ^5^ Centre for Statistics in Medicine, University of Oxford, Oxford, UK.*

|  | | ITEM | RECOMMENDATION | Section/ Paragraph |
| --- | --- | --- | --- | --- |
| 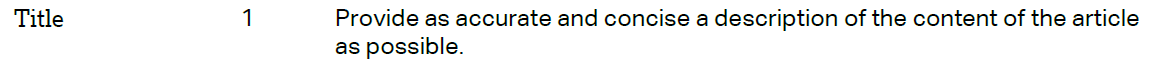 | | | title |  |
| 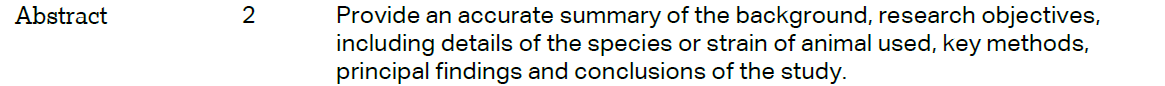 | | | abstract |  |
| INTRODUCTION | | |  |  |
| 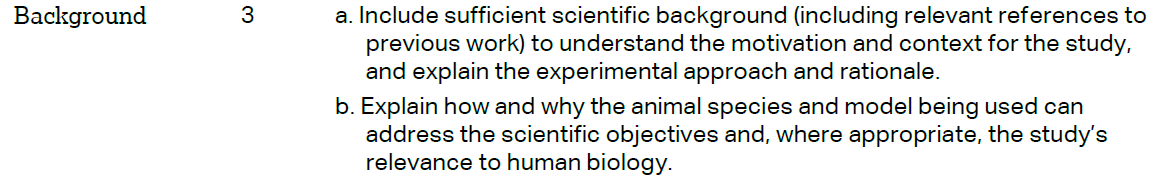 | | | Paragraphs 1-2 |  |
| 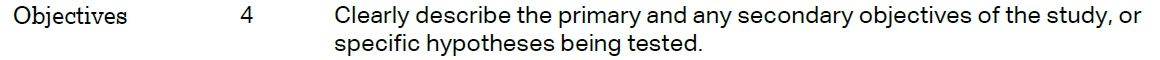 | | | Paragraph 3 |  |
| METHODS | | |  |  |
| 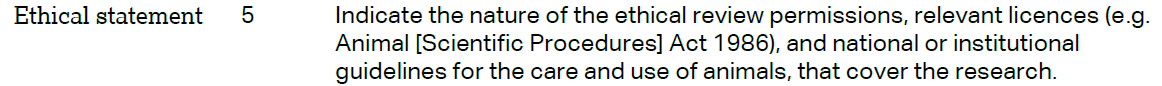 | | | Paragraph 1 |  |
| 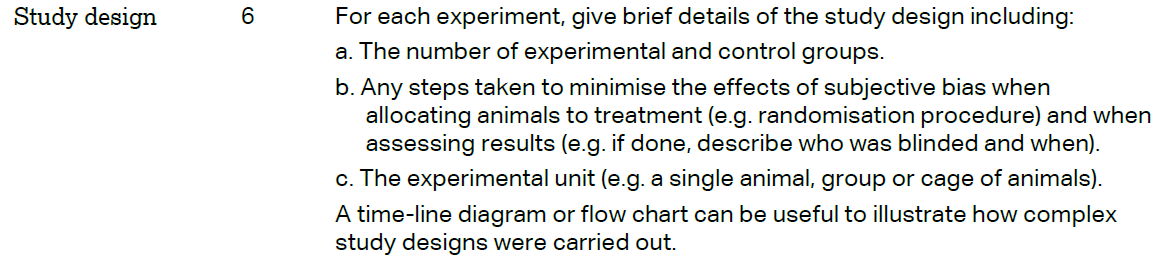 | | | Paragraphs 2-5 |  |
| 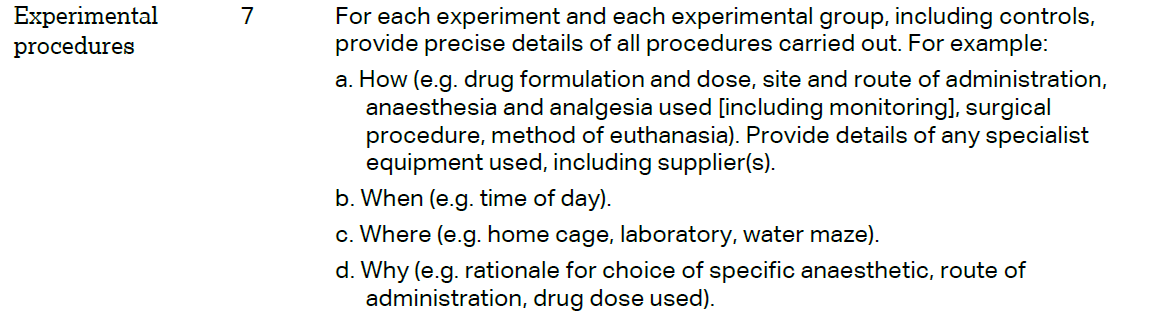 | | | Paragraphs 2-5 |  |
| 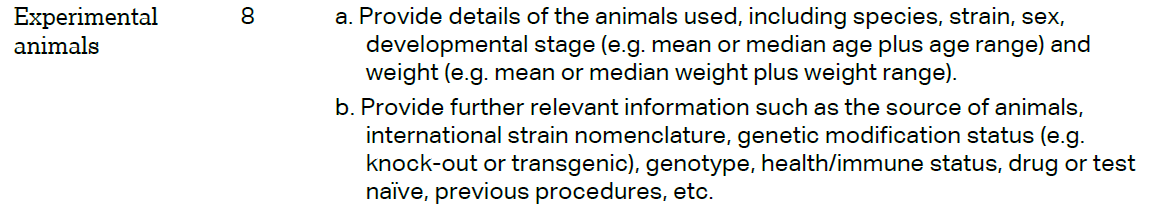 | | | Paragraphs 1-5 |  |

The ARRIVE guidelines. Originally published in *PLoS Biology*, June 2010^1^

| 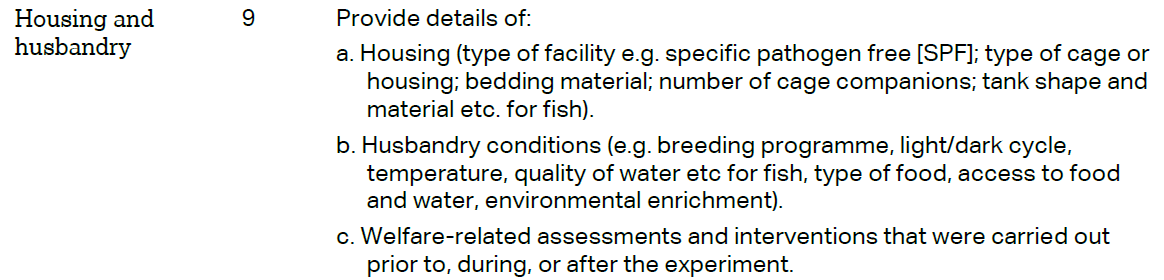 | Paragraph 1 | |
| --- | --- | --- |
| 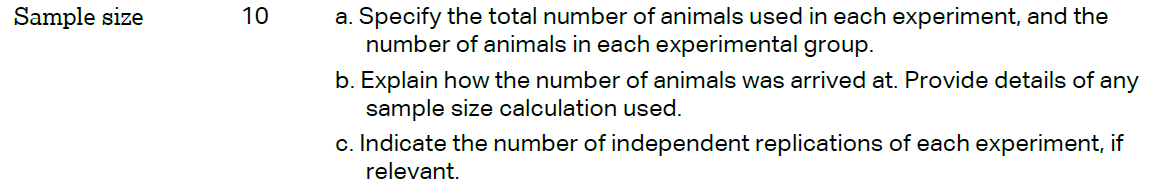 | Paragraphs 2-5 | |
| 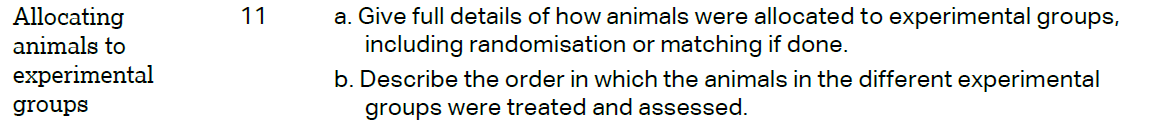 | Paragraph 2 | |
| 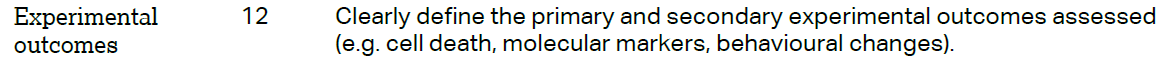 | Paragraphs 2-5 | |
| 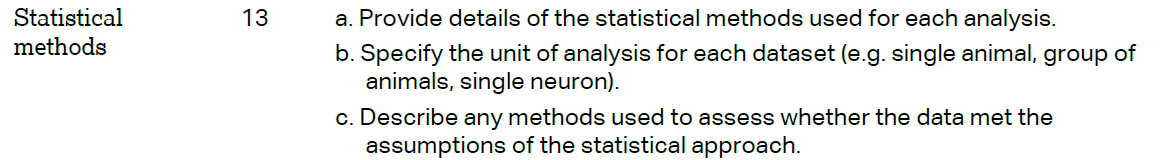 | Page 10 statistical analyses | |
| RESULTS |  | |
| 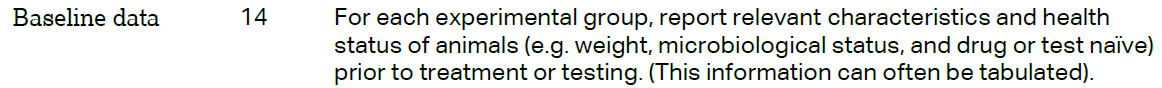 | Paragraphs 1-5 | |
| 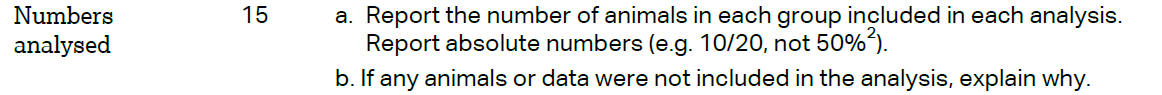 | Paragraphs 1-5 | |
| 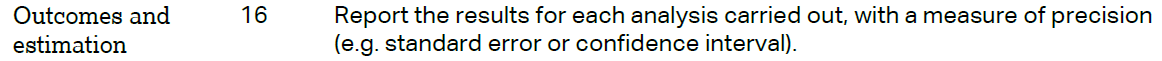 | Paragraphs 1-5 | |
| 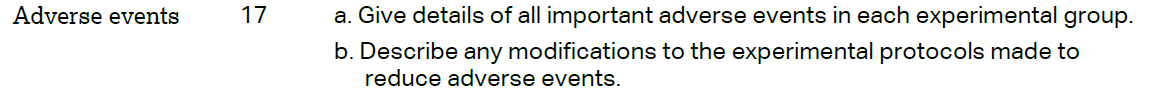 | Paragraphs 1-5 | |
| DISCUSSION |  | |
| 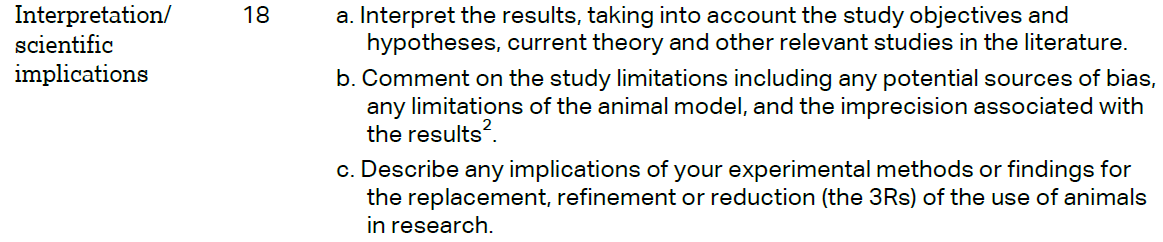 | Paragraphs 1-5 | |
| 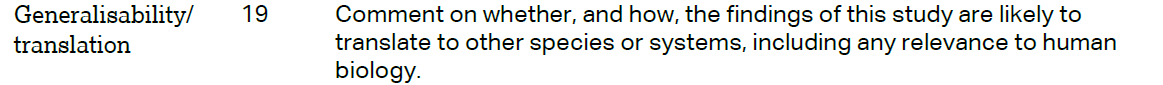 | Paragraph 6 | |
| 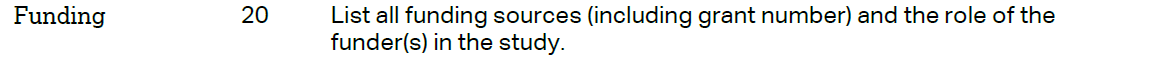 | | Page 20 acknowledgements |


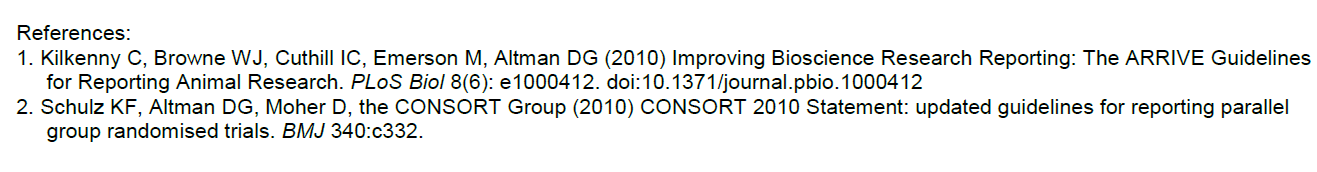

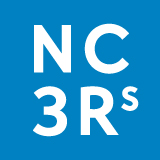

Supplement: S1 Checklist — (DOCX) [file pone.0135999.s001.docx]
