# Supplementary figures and images for: The Therapeutic Effect of Pamidronate on Lethal Avian Influenza A H7N9 Virus Infected Humanized Mice
Source: PLoS One. 2015 Aug 18;10(8):e0135999. doi: 10.1371/journal.pone.0135999 (PMC4540487; doi:10.1371/journal.pone.0135999)

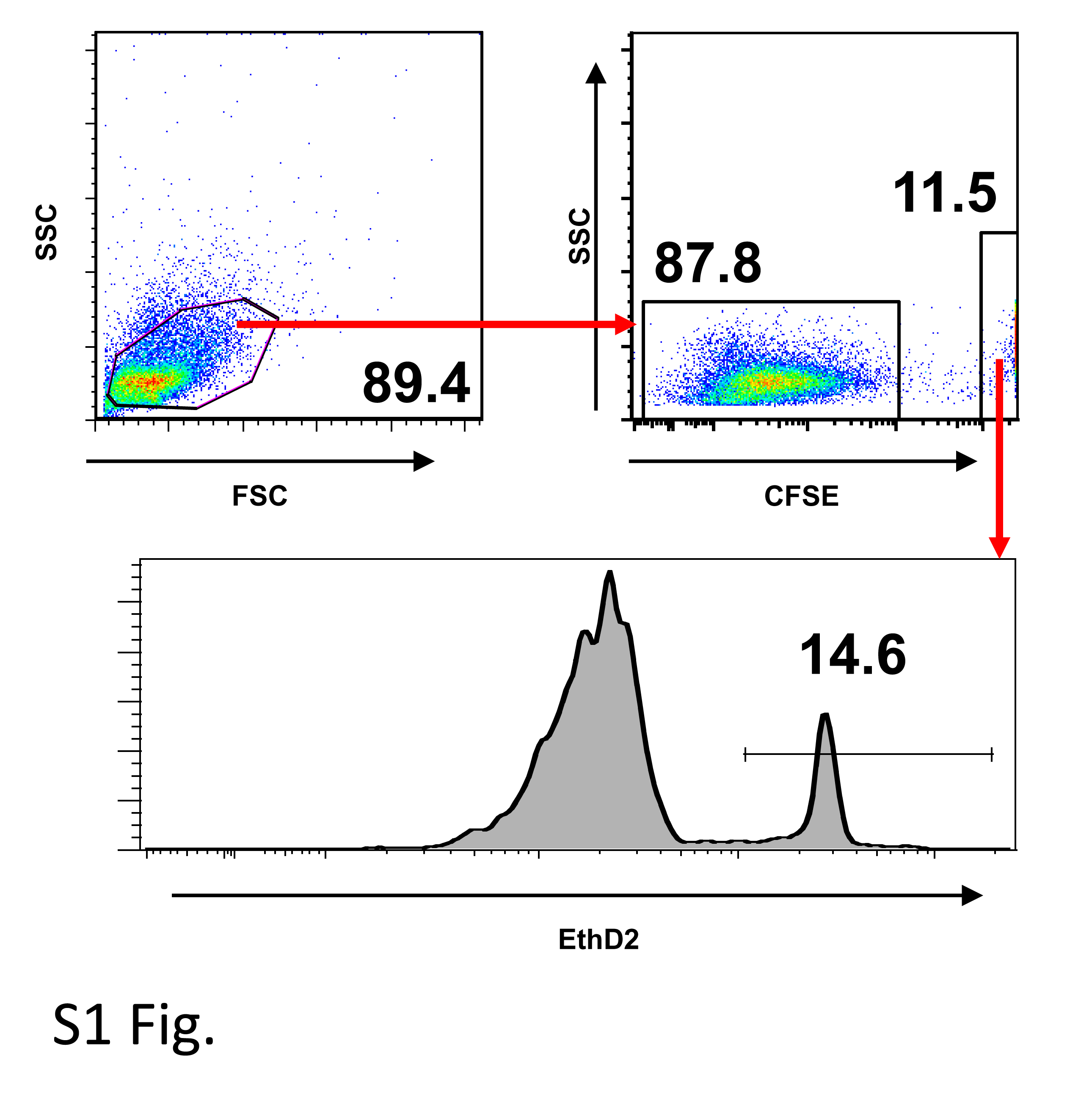

Supplement: S1 Fig — Total cells in the co-culture of Vδ2-T cells and H7N9 virus–infected MDMs were sorted by forward scatter (FSC) and side scatter (SSC) firstly. MDMs were then gated as CFSE+ population, in which EthD-2+ cells represented for dead target cells killed by Vδ2-T cells. (TIF) [file pone.0135999.s002.tif]

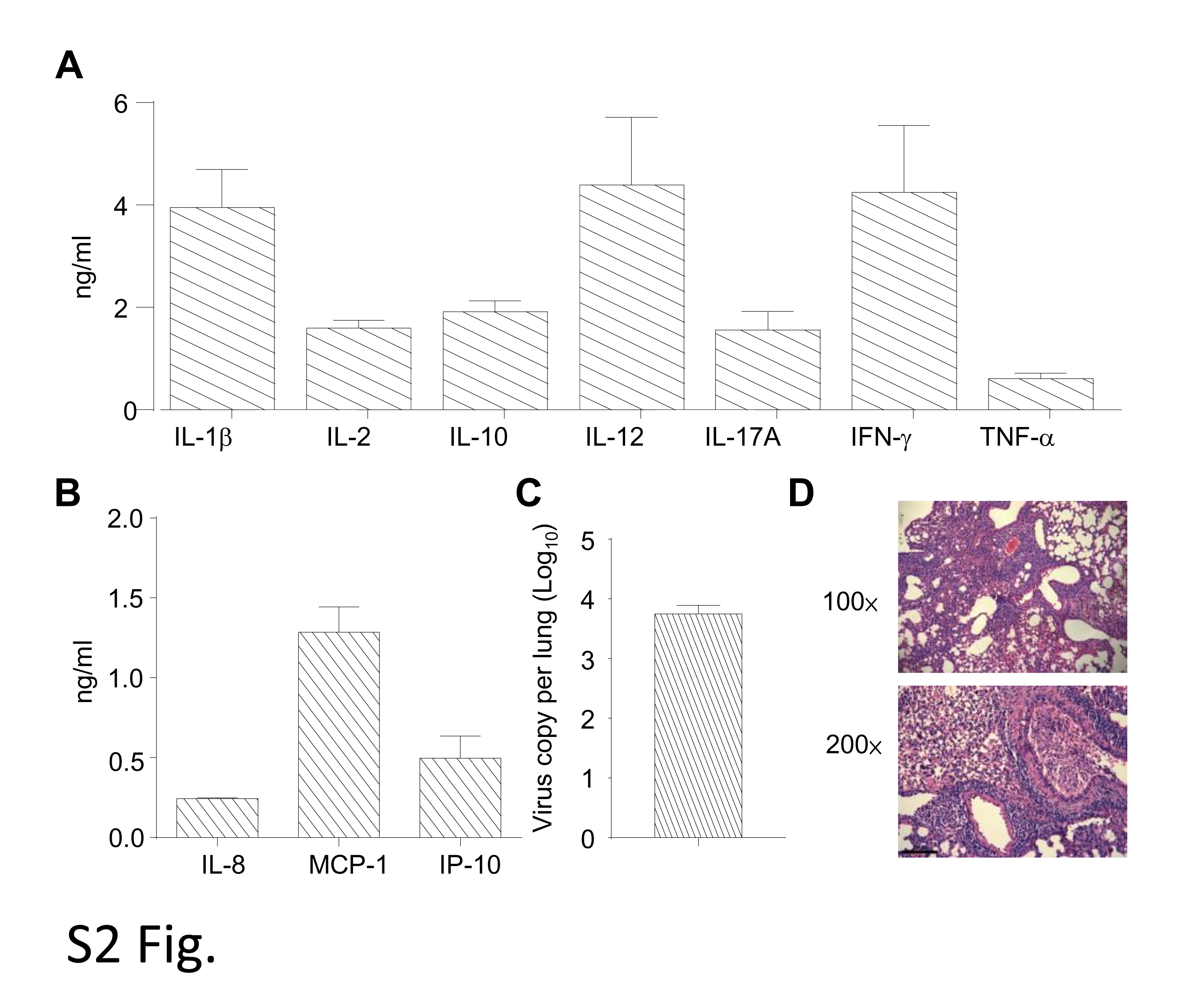

Supplement: S2 Fig — Humanized mice were infected with H7N9 virus i.n.at 106 TCID50 in 25μl saline solution on day 0. On day 3 post infection, lungs from virus-infected mice were harvested and the levels of pro-inflammatory cytokines (A) and chemokines (B) and the viral loads (C) in the supernatants of homogenized lung tissue were determined (n = 3). Representative histological sections of the lung tissues from H7N9 virus-infected mice on day 3 post infection were stained with hematoxylin and eosin. Bars, 100μm. The data are representative of three independent experiments. (TIF) [file pone.0135999.s003.tif]

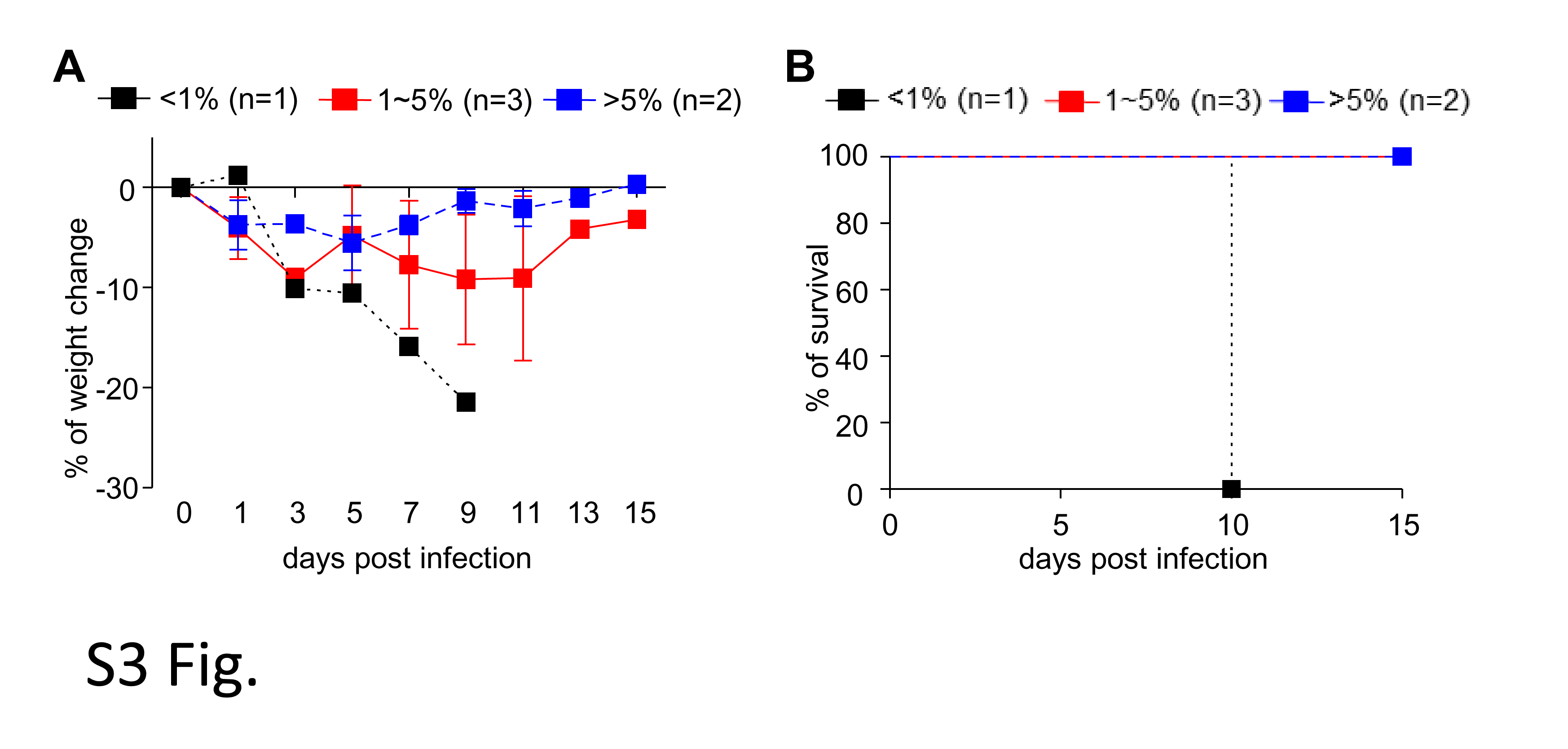

Supplement: S3 Fig — Pamidronate-treated humanized mice as shown in Fig 2 were grouped according to the initial percentage of Vδ2-T cells in peripheral CD3+ T cells. Their weight change (A) and survival (B) were shown here. (TIF) [file pone.0135999.s004.tif]
